# Energy metabolism disturbance in migraine: From a mitochondrial point of view

**DOI:** 10.3389/fphys.2023.1133528

**Published:** 2023-04-13

**Authors:** Yicheng Wang, Yongli Wang, Guangxin Yue, Yonglie Zhao

**Affiliations:** ^1^ Department of Neurology, The Third Affiliated Hospital of Beijing University of Chinese Medicine, Beijing, China; ^2^ Department of Neurology, Xiamen Hospital of Traditional Chinese Medicine, Xiamen, China; ^3^ Institute of Basic Theory for Chinese Medicine, Chinese Academy of Chinese Medical Sciences, Beijing, China

**Keywords:** migraine, mitochondrial, energy metabolism, oxidative phosphorylation, ROS, CGRP, NO

## Abstract

Migraine is a serious central nervous system disease with a high incidence rate. Its pathogenesis is very complex, which brings great difficulties for clinical treatment. Recently, many studies have revealed that mitochondrial dysfunction may play a key role in migraine, which affects the hyperosmotic of Ca^2+^, the excessive production of free radicals, the decrease of mitochondrial membrane potential, the imbalance of mPTP opening and closing, and the decrease of oxidative phosphorylation level, which leads to neuronal energy exhaustion and apoptosis, and finally lessens the pain threshold and migraine attack. This article mainly introduces cortical spreading depression, a pathogenesis of migraine, and then damages the related function of mitochondria, which leads to migraine. Oxidative phosphorylation and the tricarboxylic acid cycle are the main ways to provide energy for the body. 95 percent of the energy needed for cell survival is provided by the mitochondrial respiratory chain. At the same time, hypoxia can lead to cell death and migraine. The pathological opening of the mitochondrial permeability transition pore can promote the interaction between pro-apoptotic protein and mitochondrial, destroy the structure of mPTP, and further lead to cell death. The increase of mPTP permeability can promote the accumulation of reactive oxygen species, which leads to a series of changes in the expression of proteins related to energy metabolism. Both Nitric oxide and Calcitonin gene-related peptide are closely related to the attack of migraine. Recent studies have shown that changes in their contents can also affect the energy metabolism of the body, so this paper reviews the above mechanisms and discusses the mechanism of brain energy metabolism of migraine, to provide new strategies for the prevention and treatment of migraine and promote the development of individualized and accurate treatment of migraine.

## Introduction

Migraine is a syndrome with various neurological and non-neurological manifestations. It is the sixth leading cause of disability in the world. With the development of time, migraine is gradually ranked at the top, affecting 11% of the world’s adults’ physical and mental health, seriously affecting personal health, quality of life, and social and economic development (Murray et al., 2012; Cauchi and Robertson, 2016; Kowalska et al., 2016; Goadsby et al., 2017). Several Italian surveys have shown that migraine has a significant impact on the interpersonal relationships, emotional state, and quality of life of patients in their daily lives (Leonardi et al., 2010; Raggi et al., 2013). Therefore, the prevention and treatment of migraine has become a global problem and is of increasing concern.

The complex pathogenesis of migraine poses several difficulties for the study of migraine. Impaired cerebral energy metabolism is closely related to migraine attack threshold, and abnormal mitochondrial enzyme function is a common cause of impaired cerebral energy metabolism. Under specific environmental and triggering factors, oxidative stress and impaired cerebral energy metabolism can not only trigger migraine attacks, but can even influence the severity of migraine (Gross et al., 2019). Mitochondria are sites of energy production and release and play an important role in various tissues. They regulate the energy metabolism of brain neurons and other cells mainly through the regulation of ATP synthesis, oxidative phosphorylation reactions, tricarboxylic acid cycle (TCA), and ion homeostasis. Most of the body’s energy supply is generated through the oxidative phosphorylation pathway, or the electron transport chain (ETC) contained in the mitochondria. Oxygen consumption in the brain can account for up to 25% of the body’s total consumption, and its effective function requires a continuous supply of energy from ATP.

Abnormal energy metabolism of mitochondria can easily damage brain tissue’s normal function, so the brain and muscles are the most seriously damaged tissues in mitochondrial diseases (Roos-Araujo et al., 2014). It has been shown that the prevalence of migraine in patients with mitochondrial disease is high, suggesting that mitochondrial dysfunction may be one of the triggers for the development of migraine (Tiehuis et al., 2020). The phenomenon of cortical spreading depression (CSD) was first discovered by Leao in 1944 in experimental animals, CSD is a wave of intense depolarization of neurons and glial cells, originally described as a cortical response to noxious stimuli, manifested by negative fluctuations in cortical stability potentials and spontaneous suppression of cortical electrical activity. During the recovery of CSD, a large number of ion pumps are activated and oxygen consumption and metabolism increase, leading to hypoxia in brain tissue. In hypoxia, the ETC and oxidative phosphorylation pathways are impaired, enzyme activity in the ETC is inhibited, ATP synthesis is blocked, and excess oxygen radicals are generated, causing damage to the mitochondria, which in turn leads to cell damage. Nitric oxide (NO) is a key factor in the development of migraine and other headaches and is thought to be the trigger point for primary headaches, playing a key role in the development of migraine (Olesen, 2008). Calcitonin gene-related peptide (CGRP) is a multifunctional neuropeptide. It plays an important role in the pathophysiology of migraine by modulating neurogenic inflammation and regulating nociceptive afferents (Russell et al., 2014; Russo, 2015), CGRP and its receptors are being used as promising targets for the treatment of migraine (Edvinsson, 2015). Experimental studies have shown that changes in neurotransmitters and vasoactive substances caused by CSD play an important role in migraine attacks and that the occurrence of CSD can cause changes in the release of NO in plasma and the levels of vasoactive peptides such as CGRP (Read et al., 2000). High concentrations of NO and CGRP can further affect the activity of various components of the mitochondrial respiratory chain, resulting in damage to the mitochondrial respiratory chain, reduced ATP production, and excessive reactive oxygen species (ROS) production, leading to oxidative stress and thus causing neuronal dysfunction, which can lead to migraine (Benemei et al., 2013). Mitochondrial dysfunction and excessive ROS production can also affect the expression of NO and CGRP, and the above factors interact with each other to eventually lead to the development of migraine.

In this paper, oxidative phosphorylation, tricarboxylic acid cycle, oxidative stress, NO and CGRP, which are closely related to migraine, are used as entry points to explore the mechanism of cerebral energy metabolism in migraine and provide new ideas for the treatment of migraine.

## Cortical spreading depression and migraine

CSD is one of the factors in the pathogenesis of migraine. Studies have shown that patients with migraine have the transmission pattern of CSD, which activates the trigeminovascular system (TVS) pathway (Unekawa et al., 2015). CSD can induce significant changes in neurovascular responses, including the release of NO, while high concentrations of NO can significantly inhibit the activity of many components in the mitochondrial respiratory chain, resulting in damage to the mitochondrial respiratory chain. Mitochondrial damage in any part of the brain may lead to mitochondrial energy deficiency, while migraine is a response to brain energy deficiency or oxidative stress levels exceeding antioxidant capacity (Fila et al., 2019; Gross et al., 2019). Studies have shown that CSD can be an inducer of ROS formation in the cerebral cortex, meninges and trigeminal ganglia. ROS may be involved in the formation of central and peripheral sensitization by regulating protein kinase activity, altering glutamatergic nerve transmission, mediating neurogenic inflammation, and regulating ion channels, such as transient receptor potential channel V1 (TRPV1) (Meents et al., 2010). ROS can also directly activate pain receptors and promote peptidergic nerves to release migraine mediator CGRP. CGRP can act on vascular smooth muscle cells, vascular endothelial cells, and trigeminal ganglion nerve cells, resulting in corresponding vasodilation, further causing the attack of migraine. A critical role in the regulation of cortical susceptibility to CSD is played by ROS/transient receptor potential A1 (TRPA1)/CGRP signalling. Inhibition of ROS and deactivation of TRPA1 channels may have therapeutic benefits in the prevention of stress-induced migraine via CGRP (Jiang et al., 2019). During CSD episodes, it leads to hypoxia in the brain, which impairs the normal function of mitochondria and eventually leads to oxidative stress, which can also further induce CSD (Borkum, 2021).

Some studies have shown that the increase of intracellular Ca^2+^ concentration in astrocytes leads to vasoconstriction during CSD, which is mediated by phospholipase A2, an arachidonic acid metabolite (Metea and Newman, 2006). A high concentration of intracellular Ca^2+^ enhances peripheral pain, while a low concentration of intracellular Ca^2+^ inhibits peripheral pain. The main mechanism of intracellular Ca^2+^ blocking is through mitochondria, and mitochondrial dysfunction can lead to pain hypersensitivity. Mitochondrial calcium overload can depolarize the mitochondrial membrane and increase ROS, resulting in mitochondrial dysfunction, and affects ATP production through the Mitochondrial Permeability Transition Pore (mPTP), Cytochrome c (Cyt-c), Nitric Oxide Synthase (NOS), ETC, Complex I and Complex IV (Brookes et al., 2004; Xu et al., 2020a).

**FIGURE 1:**
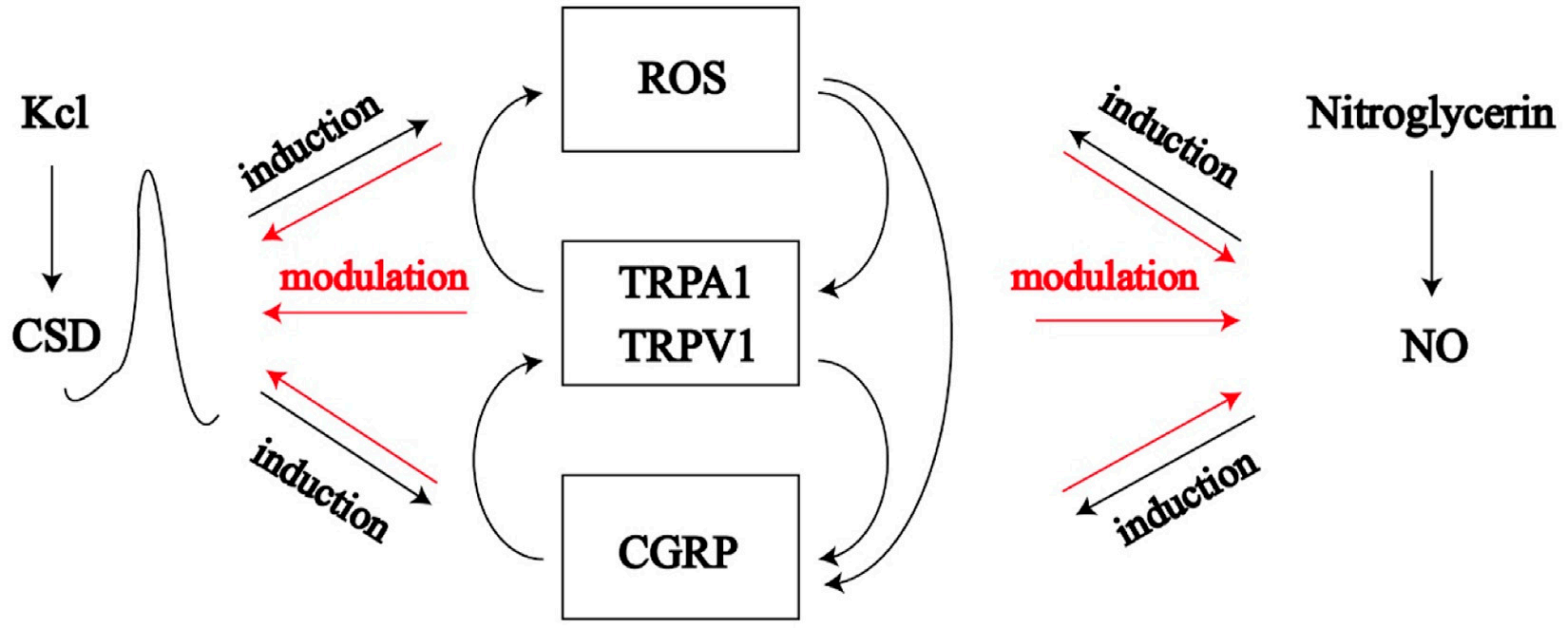
Cortical spreading depression induced by potassium chloride and nitric oxide release induced by nitroglycerin can affect ROS/TRPA1/TRPVA/CGRP pathway, which forms positive feedback with CSD and NO, which leads to energy metabolism disorder and migraine.

CSD, cortical spreading depression; ROS, reactive oxygen species; NO, nitric oxide; TRPA1, transient receptor potential ankyrin 1; TRPV1, transient receptor potential vanilloid 1; CGRP, calcitonin gene and related peptide.

CSD upregulates the expression of cyclooxygenase-2(COX-2), tumor necrosis factor-α(TNF-α), interleukin-1beta (IL-1β), and proteins encoded by the ghrelin and matrix metalloproteinases (MMPs). Activation of MMPs can open the blood-brain barrier and alter the extracellular fluid composition of the cerebral cortex, increasing K^+^, H^+^, NO, and epinephrine, which sensitize or excite the ipsilateral trigeminal vascular nerve fibers (Figure 1) (Iadecola, 2002).

## Relationship between oxidative phosphorylation and migraine

Mitochondria have multiple functions, an important one being the production of ATP through oxidative phosphorylation (eukaryotes). Experimental results suggest that CSD may exacerbate brain mitochondrial damage under hypoxic conditions (Li et al., 2011). Mitochondrial damage includes changes in mitochondrial respiratory function and mitochondrial membrane potential, and hypoxia can lead to cell death and induce migraine (Ouyang et al., 2006). In the mitochondria, the process of ATP production depends on the cellular respiratory function. 95% of the energy needed for cell survival is provided by the mitochondrial respiratory chain, which includes two processes: TCA and oxidative phosphorylation. The oxidative phosphorylation process is mainly involved in five molecular complexes with enzyme activity located on the mitochondrial inner membrane, namely, complex I, complex II, complex III, Complex IV, and complex V, namely, ATP synthase (Kobayashi et al., 2020).

Complex I (NADH ubiquinone oxidoreductase/NADH dehydrogenase) has a redox module and is a classical L-shaped structure, including hydrophobic and hydrophilic structures. It is the main electron portal of the mitochondrial electron transport chain (mETC) and provides up to 40% protons for the formation of mitochondrial ATP. Under pathological conditions, complex I am the main source of reactive oxygen species. It was found that both PACAP-38 (pituitary adenylate cyclase-activating polypeptide-38) and PACAP (6–38) treatment resulted in significant downregulation of complex I subunit B6 expression in primary cultured trigeminal ganglion cells (Takács-Lovász et al., 2022), and metabolic changes and mitochondrial dysfunction, such as reduced complex I, III, IV and citrate synthase activities, were detected in migraine patients (Gross et al., 2019).

Complex II (succinate-ubiquinone oxidoreductase/succinate dehydrogenase) exists in various aerobic organisms and is a complete membrane protein complex in the tricarboxylic acid cycle and aerobic respiration. Studies have shown that pro-apoptotic compounds, such as various anticancer drugs. Fas Ligand (FasL), or TNF-α, can induce a decrease in cytoplasmic and mitochondrial pH. These pH changes lead to the dissociation of SDHA and SDHB subunits from complex II, resulting in partial inhibition of complex II activity without any damage to SDH response. This specific inhibition leads to complex II uncoupling, superoxide production, and apoptosis (Lemarie et al., 2011). Riboflavin is required for the conversion of oxidized glutathione to the reduced form and mitochondrial respiratory chain, as complex I and II contain flavoprotein reductase and electron transferring flavoprotein (Plantone et al., 2021). Riboflavin deficiency has been shown to impair the oxidative state of the body, and some experiments have also shown that riboflavin can be effective in treating migraines (Thompson and Saluja, 2017).

Complex III (cytochrome c reductase/cytochrome bc1 complex) is the central component of the respiratory chain, which transfers electrons from coenzyme Q (transfer from complex I and complex II) to cytochrome c (within complex IV) and contributes to the production of proton gradients (mitochondrial inner membrane). Migraine prevention can be achieved by applying highly specific inhibitors of oxidant production at the ubiquinone position of complex I or complex III (Orr et al., 2015). These inhibitors do not affect electron transport or ATP production, but they do reduce ROS. This approach prevents oxidative stress from reverse electron transport, but presumably does not affect the consumption of antioxidant defences by nicotinamide nucleotide transhydrogenase (NNT), which operates in the reverse mode (Borkum, 2021).

Complex IV (cytochrome c oxidase) is the last electron acceptor of the respiratory chain, which participates in the reduction of oxygen to water molecules and transfers protons in the mitochondrial matrix to the intermembrane space, which is also helpful to produce the transmembrane proton gradient difference. Complex IV has been shown to bind to NO. NO isolation may exist in the blood vessels rich in complex IV, thus preventing vasodilation (Torres et al., 1995). Complex Ⅳ is the main target of gas signaling molecules to inhibit mitochondrial respiration. NO, carbon monoxide (CO) and hydrogen sulfide (H2S) can all reduce oxygen consumption and ATP production through complex Ⅳ pathway (Cooper and Brown, 2008). The mitochondrial dysfunction induced by nitroglycerin (GTN) was associated with abnormal levels of Bax, Bcl-2, cytochrome C oxidase and ROS, and these changes were attenuated by valproate treatment. As Bax, Bcl-2 and ROS are closely related to cell apoptosis, the abnormal Bax, Bcl-2 and ROS levels in study suggest that migraine may also be related to neurocyte apoptosis (Li et al., 2016).

The final enzyme of the oxidative phosphorylation pathway is the ATP synthase complex, which can use the transmembrane proton electrochemical potential energy formed by mETC to drive ADP to combine with Pi to form ATP. ATP synthase complex is a kind of protein complex that can exchange mitochondrial ADP and other inorganic salts with ATP. The ATP synthase complex is composed of F1F0-ATP synthase and two members of the mitochondrial solute carrier (SLC) protein family, namely, adenine nucleotide transferase (ANT) and mitochondrial phosphate carrier (PiC). Among them, the SLC25A3 gene is an important part of the PiC gene. It is found that the expression of PiC is closely related to the expression of SLC25A3 (Bhoj et al., 2015). ANT includes three subtypes, namely, SLC25A4 (ANT1), SLC25A5 (ANT2), and SLC25A6 (ANT3), while SLC25A3 mediates mitochondrial absorption and is responsible for the exchange between ATP and ADP.

Studies have shown that the dimer of mitochondrial ATP synthase is very important for the formation of mPTP (Giorgio et al., 2013). The c subunit of mitochondrial ATP synthase may be necessary for mPTP-dependent mitochondrial breakage and cell death (Bonora et al., 2013). F1F0-ATP synthase is a reversible enzyme, when calcium flux out of the matrix is positive, the proton gradient is reinforced, supporting the production of ATP. Conversely, when the flux is negative, the proton gradient is compromised. If too many protons accumulate in the matrix, the F0F1-ATP synthase reverses and the enzyme consumes ATP rather than produces it (Xu et al., 2020b). CSD is one of the pathogenesis of migraine. During CSD attacks, Ca^2+^ enters mitochondria in large quantities, thus affecting F0F1-ATP synthase, so F0F1-ATP synthase is a new direction to study the pathogenesis of migraine. A feedforward loop will be formed among Ca^2+^, ROS, and mPTP (Qi and Shuai, 2016). That is, the accumulation of Ca^2+^ in mitochondria can promote the production of ROS (Bertero and Maack, 2018), they act together to regulate the opening of the mPTP (Kovac et al., 2017). Among them, Ca^2+^ enters the mitochondria through the mitochondrial calcium uniporter (MCU) complex and leaves the mitochondria through Na^+^/Ca^2+^ exchanger (mNCX), H^+^/Ca^2+^ exchanger (mHCX), and mPTP. The transient opening of mPTP can quickly release Ca^2+^.

### Calcium ion

Ca^2+^ homeostasis in mitochondria is achieved through mNCX, mHCX, MCU complex, and the mPTP (Agarwal et al., 2017). Excessive Ca^2+^ in mitochondria may induce oxidative stress by depolarizing mitochondria, increasing the activity of complex I and II, and impairing mitochondrial function. Mitochondria play an important role in regulating Ca^2+^ homeostasis. The absorption of Ca^2+^ by mitochondria can buffer the concentration of Ca^2+^ in and out of the cytoplasm, form intracellular Ca^2+^ signal, and stimulate ATP production. When too much Ca^2+^ is ingested, calcium phosphate deposits are formed in the mitochondria, which reduces the production of ATP and leads to mitochondrial dysfunction.

CaV2.1 channels are calcium channels located in the presynaptic membrane and play an important role in communicating between neurons by controlling neurotransmitter release, while presynaptic Cav2 channels might be expected to drive CGRP release associated with migraine, high-voltage-activated and canonical postsynaptic Cav1 channels and low-voltage-activated Cav3 channels have both been found to regulate CGRP release in the trigeminal ganglion. Ca^2+^, potassium and sodium levels are all altered in the course of a migraine. This has led scientists to argue that migraine is a channelopathy (Antunes et al., 2022). CSD is a pathophysiological phenomenon that may be a contributor to brain damage in migraine. The researchers found that topical application of a cortical ionophore increased the rate of CSD propagation and that a higher dose of this compound induced CSD, suggesting a role for Ca^2+^ influx into cells in CSD (Torrente et al., 2014).

### Mitochondrial permeability transition pore

The mitochondrial permeability transition pore is a complex located on the mitochondrial membrane, which was previously thought to be composed of voltage-dependent anion channels (VDAC) in the outer membrane of the mitochondria, ANT in the mitochondrial inner membrane, and cyclophilin D (CypD) in the matrix (Haleckova et al., 2022). Stimulation of primary cultured trigeminal ganglion cells with capsaicin can mimic migraine attacks *in vitro* (Wang et al., 2016), and studies have revealed transcriptional upregulation of cytochrome c oxidase subunit IV, Mic60/Mitofilin, and VDAC1, which implies induction of mitochondrial biogenesis to compensate for the loss of mitochondria (Shibata et al., 2020). Other studies have suggested that mPTP is formed by dimers of F1F0-ATP synthase, this is called the F1F0-ATP synthase dimer model for the formation of mPTP. However, Bonora et al. believe that the c subunit of F1F0-ATP synthase is the key component of mPTP. Studies have shown that the c subunit ring of purified and reconstructed F1F0-ATP synthase forms a voltage sensitive channel, which leads to rapid and uncontrolled depolarization of the mitochondrial inner membrane in cells. High concentration of Ca^2+^ in the mitochondrial matrix for a long time enlarged the c subunit loop and dissociated it from the CypD/ciclosporin A (CsA) binding site of the F1F0-ATP synthase F1 subunit. According to the latest study by Bonora (Bonora et al., 2017), the opening of the permeability transition pore complex requires the dissociation of the F1F0-ATP synthase dimer and includes the c ring of the F1F0-ATP synthase. Therefore, it is considered that the c subunit channel of highly regulated F1F0-ATP synthase may be mPTP, which is called the F1F0-ATP synthase monomer and c subunit ring model. Recently, Giorgio et al. have found that the binding of Ca^2+^ to the β subunit of mitochondrial F1F0-ATP synthase leads to the transition of mitochondrial permeability. In any case, these studies provide convincing evidence that F1F0-ATP synthase is necessary for mPTP function.

Under physiological conditions, mPTP opens periodically and non-selectively to allow water and small molecules with a relative molecular weight of <1.5 × 10^3^ kDa to pass through. This maintains the electrochemical balance in the mitochondria, while protons can freely pass through the inner mitochondrial membrane, creating a potential difference inside and outside the mitochondrial matrix and forming a balanced mitochondrial membrane potential. Under various exogenous pathological stimuli, mPTP opened explosively, allowing substances with a relative molecular weight of more than 1.5 × 10^3^ kDa to pass non-selectively, resulting in the collapse of mitochondrial membrane potential, the decoupling of oxidative phosphorylation, and the inhibition of ATP production. Because the surface area of the inner membrane of the mitochondria is significantly larger than that of the outer membrane, the swelling of the mitochondrial matrix caused by the opening of mPTP will lead to the rupture of the outer membrane of the mitochondria, promote the release of Cyt-c and other pro-apoptotic factors into the cytoplasm and initiate endogenous apoptosis.

Programmed cell death (PCD) refers to the process of orderly removal of non-essential cells, specialized cells, or injured cells by suicide under the control of related genes and signal pathways. The previously discovered PCD types include intrinsic and extrinsic apoptosis (Galluzzi et al., 2012), autophagy, necroptosis, parthanatos, pyroptosis, and ferroptosis. GTN-mediated increases of pain intensity, apoptosis, death, cytosolic ROS, mitochondrial ROS, caspase-3, caspase-9, cytosolic Ca^2+^ levels, and cytokine generations (TNF-α, IL-1β, and IL-6) in the TG of transient receptor potential melastatin 2 (TRPM2) wild-type mouse were further increased by the TRPM2 activation (Yazğan and Nazıroğlu, 2021). It is worth noting that the emergence of mitochondrial permeability transition is a necessary condition for the occurrence of apoptosis, autophagy and necroptosis (Vanden Berghe et al., 2014), the knockout of CypD or the use of its inhibitor CsA can effectively resist PCD, suggesting that the mPT phenomenon may be mediated by a specific channel composed of a series of macromolecular proteins including CypD, which is an important target for mitochondrial regulation of PCD process confirmed by a large number of studies in the future.

### Cyclophilin D

CypD is a mitochondrial matrix protein encoded by Peptidyl-prolyl cis-trans isomerase F(PPIF). It is a member of the macrocyclic protein gene family. The complete CypD protein consists of 207 amino acids (22 kDa). Its most prominent feature is the cyclophilin domain of 109 amino acids, which endows most cyclophilin with conservative prolyl isomerase activity. CypD is the only molecule genetically identified to regulate the opening of mPTP. Hafner et al. reported that the decrease of NAD+/NADH ratio will cause a decrease in silent mating-type information regulation 2 homolog 3 (SIRT3) activity, while the decrease of SIRT3 activity can increase the acetylation level of CypD, which can induce the open and membrane potential depolarization of mPTP by inducing the acetylation of CypD (Yang et al., 2015). Under physiological conditions, the transient opening of mPTP mediated by CypD can lead to slight changes in mitochondrial membrane potential, which does not have any adverse effect on cell viability, and can reduce excessive metabolites and ions (especially Ca^2+^) in mitochondria, to avoid mitochondrial swelling, prevent the release of pro-apoptotic factors, and finally maintain the integrity of mitochondria. mPTP is an adjustable non-selective protein channel through which water and solute, including VDAC through the mitochondrial outer membrane (OMM) and ANT through the mitochondrial inner membrane (IMM) (Gutiérrez-Aguilar and Baines, 2015; Briston et al., 2017). Under pathological conditions, the permeability transition pores continue to open under the stimulation of oxygen free radicals or Ca^2+^, resulting in mitochondrial swelling, the release of Cyt-c, and consequently caspase-3 activation, resulting in a series of mitochondria-mediated apoptotic cascade responses (Eloy et al., 2012). Stimulation of cells by H_2_O_2_ can simulate oxidative stress in migraine *in vitro*, a model of CypD protein expression or high expression in endothelial cells was established through gene silencing or cloning. The cell apoptosis rate of the CyPD low expression group was significantly lower than that of the control group, the apoptosis rate of the CyPD high expression group was significantly higher than that of the control group. CypD protein could increase oxidative stress and cause endothelial cell injury and apoptosis (Peng et al., 2015). It was shown that CypD could bind to the oligomycin sensitivity-conferring protein (OSCP) of the lateral stalk of ATP synthase, which is composed of OSCP, F6, b, and d subunits (Giorgio et al., 2009), when the OSCP on the side stalk of ATP synthase is reduced, it can increase the sensitivity of mPTP to Ca^2+^ and also lead to the destabilization of ATP synthase.

It was found that the interaction between TNF receptor associated protein 1 (TRAP1) and F1F0-ATP synthase increased its enzyme activity and inhibited the opening of mPTP. TRAP1 and CyPD affect cell bioenergy characteristics and survival in noxious conditions by competing with each other for their binding to F1F0-ATP synthase OSCP subunits, which plays an important role in pathophysiological conditions such as tumor transformation or adaptation to hypoxia (Cannino et al., 2022).

### Oligomycin sensitivity-conferring protein

OSCP contains 180–190 amino acids, encoded by the nuclear ATP50 gene, its molecular weight is about 23 kDa and plays a key role in the assembly of ATP synthase. It occurs in a modular manner, preventing the formation of OSCP that can depolarize the membrane or waste intermediates of ATP (Rühle and Leister, 2015). Mitochondrial F1F0-ATP synthase is a complex V on the respiratory chain of the mitochondrial inner membrane and a 600 kDa multi-subunit complex, which is mainly composed of two parts: one is the spherical catalytic part, that is, the F1 part protruding from the inside of the membrane, including a, β, γ, and other subunits; the other part is the proton transport part, that is, the F0 part embedded in the membrane, including a, c, e, A6L, f, g, and other subunits, in which c subunits form c-ring (Giorgio et al., 2009). There is also a peripheral stem attached to the outer side of F1 and F0, consisting of OSCP, F6, b and d subunits, OSCP is located at the tip of F1 and is the binding target of CyPD. both F1 and F0 are connected through the peripheral stem and catalyze the synthesis of ATP.

Studies have shown that the binding ability of TRAP1 and OSCP exceeds that of CyPD and OSCP, and TRAP1 can also isolate CyPD from F1F0-ATP synthase, both of which are non-mutually exclusive. We believe that OSCP acts as a hub to fine-tune the enzyme activity of F1F0-ATP synthase and its conversion to PTP by interacting with different protein regulatory factors. The combination of OSCP and different partners will produce specific biochemical results, thus optimizing biological output to adapt to changes in environmental conditions.

Some studies have shown that honokiol treatment can increase the expression of SIRT3 nearly twice and further increase its activity. The Increased SIRT3 activity and mitochondrial SIRT3 substrate, which is related to the decrease of acetylation of OSCP. ATP synthase F1 proteins α, β, γ, and OSCP contain SIRT3-specific reversible acetyllysine, which is evolutionarily conserved and binds to SIRT3. OSCP was further studied and it was found that OSCP lysine 139 was a nutritionally sensitive target for SIRT3-dependent deacetylation (Vassilopoulos et al., 2014). To further study the function of the OSCP protein, HAP1- ΔOSCP, a specific knockdown OSCP monoclonal cell, was screened from human HAP1 cells. Compared with HAP1-WT cells, HAP1- ΔOSCP grew slowly; the copy number of mitochondrial DNA decreased by 30%; except for complex II, the protein levels of complex I, complex III, and complex IV all decreased, and the level of oxidative phosphorylation decreased significantly (He et al., 2017).

### Bcl-2 family

The Bcl-2 family includes two types of members, one is Bcl-2 and Bcl-xl which inhibit apoptosis, and the other Bax, Bak, Bcl-xs, Bad, Bid, and Bik which promote apoptosis.

Studies have confirmed that the Bcl-2 family regulates apoptosis mainly by regulating the opening of mPTP, in the experimental system of isolating mitochondria, Bcl-2 can inhibit the disintegration of mitochondrial membrane potential induced by many factors; some studies have also proved that Bcl-xl can inhibit the redistribution of Cyt-c and the depolarization of mitochondrial membrane potential, thus inhibiting apoptosis (Vander Heiden et al., 1997). Bax can interact with ANT, an important component of mPTP, to form pores in the mitochondrial membrane, while Bcl-2 can prevent this action and inhibit pore formation. In addition, the expression of Bcl-2 is related to the benzodiazepine receptor, another component of mPTP, indicating that Bcl-2 itself can regulate the opening of mPTP. Overexpression of Bcl-2 can also inhibit the open inducer of mPTP, thus inhibiting apoptosis.

Some studies have shown that PTP is mediated by the opening of the permeability transition pore complex (PTPC). PTPC is a widely used supramolecular entity assembled at the junction of the mitochondrial membrane, which is composed of CypD, VDAC, ANT, and so on. Pro-and anti-apoptotic members of the Bcl-2 family, including Bax, Bid, Bcl-2, and Bcl-xl, have been shown to bind to PTPC and thus regulate its function (Vander Heiden et al., 2001). Bcl-xl is the main anti-apoptotic protein in the adult brain. Studies have shown that Bcl-xl directly interacts with the β-subunit of F1F0-ATP synthase to reduce ion leakage in F1F0-ATP synthase, thus increasing the transport of H^+^ by F1F0 in the process of F1F0-ATP synthase activity (Alavian et al., 2011). Bcl-xl also enhances the exchange of metabolites between mitochondria and cytoplasm through interaction with VDAC, helping to prevent the release of death-promoting factors. The enhancement of ATP production by the mitochondrial inner membrane F1F0-ATP synthase complex requires VDAC to remain open to release newly synthesized ATP into the cytoplasm. Neurons overexpressing Bcl-xl had higher levels of ATP, while cells with depleted or suppressed endogenous Bcl-xl had lower levels of ATP. Pro-apoptotic proteins Bax and Bak accelerate the opening of VDAC, while anti-apoptotic protein Bcl-xl shuts down VDAC by directly binding to VDAC. Bax and Bak allow cytochrome c to pass through VDAC from liposomes, but Bcl-xl prevents cytochrome c from passing through (Shimizu et al., 1999). Once Bax and Bak are activated, it promotes the release of cytochrome c and the division of mitochondria, resulting in the activation of apoptotic protease activating factor-1 (apaf-1) into apoptosomes and the activation of caspase-9 to activate caspase-3. It has been shown that stimulation of the dura mater of C57BL/6 mice with inflammatory soup leading to migraine attacks can initiate a programmed cell death pathway that activates total caspase-1 and converts it to active cleaved caspase-1, which cleaves and activates total caspase-3 into cleaved caspase-3 to induce apoptosis (Wang et al., 2022). Bcl-2 is located in the outer membrane of mitochondria, which can bind with Bax to form a Bcl-2/Bax heterodimer, prevent Bax from inserting into the outer membrane of mitochondria, and protect cells from apoptosis. Bcl-2 and Bax are often co-expressed in tissues and cells. when Bcl-2/Bax increases, mPTP shuts down, which promotes cell survival and otherwise leads to cell death. Parthenolide (PTL) is a sesquiterpene lactone found in large quantities in the leaves of feverfew, which possesses anti-inflammatory, anti-migraine and anti-cancer properties. PTL was shown to significantly increase the Bcl-2/Bax ratio (Ren et al., 2019). The c-conformation of ANT (when ADP binds to the cytoplasmic side) is more conducive to the opening of mPTP, while the m-conformation of ANT (when ATP binds to the matrix side) is more conducive to the closed state of mPTP. Therefore, Atractyloside can stabilize the c-configuration of ANT and promote mPTP, while bongkrekic acid (Henderson and Lardy, 1970) can stabilize the m-configuration of ANT and inhibit mPTP.

### Mitochondrial membrane potential

Under normal circumstances, the outer mitochondrial membrane has high permeability, while the permeability of the inner mitochondria membrane is relatively low. The mitochondrial membrane potential is caused by the asymmetric distribution of electrons on both sides of the inner mitochondria membrane. The low permeability and electrochemical proton gradient of the inner mitochondria membrane are the basis for maintaining the mitochondrial membrane potential, while the normal mitochondrial membrane potential is necessary for mitochondrial function (Tsujimoto and Shimizu, 2007). CaV 2.1 voltage-gated calcium channels (VGCCs) are highly expressed by cerebellar neurons, and their dysfunction is associated with human disorders such as familial hemiplegic migraine. The researchers are studying leaner and tottering mice that carry autosomal recessive mutations in the gene coding for the α1A pore-forming subunit of CaV 2.1 VGCC. Excessive leaner cerebellar granule cell (CGC) death begins soon after postnatal day 10. Calcium homeostasis and mitochondrial membrane potential were also changed in the CGC of leaner mice (Bawa and Abbott, 2008).

Under physiological conditions, the mitochondrial membrane potential oscillates to a small extent. Under pathological conditions, metabolic stress occurs, and when the balance between ROS production and ROS clearance is disrupted, the mitochondrial network of the whole cell is locked in a low-frequency, high-amplitude oscillation mode (Aon et al., 2008). The opening and closing of mPTP are affected by many factors, in addition to free radicals, Ca^2+^, and other factors, there are some regulatory proteins: peripheral-type benzodiazepine receptor (PBR) is an important component and regulatory protein of mPTP. Mitochondrial inner membrane anion channel (IMAC) inhibitors can block mitochondrial oscillation, and PBR ligands can also reduce mitochondrial oscillation. PBR is composed of VDAC, ANT, and translocator protein (TSPO). The binding of PBR and ligand triggers the conformational change of mPTP, which induces the decrease of mitochondrial membrane potential, activation of caspase-3, and the increase of cell permeability, which leads to the release of apoptotic factors.

The reasons for the decrease of mitochondrial membrane potential have the potential to convert F1F0-ATP synthase to ATP hydrolase in theory (Diez et al., 2004). In this process, the function of ANT in the mitochondrial inner membrane also changes with the function of the F1F0-ATP synthase. Under normal physiological conditions, ANT transports the ADP in the cytoplasm to the mitochondria and transports the ATP synthesized by F1F0-ATP synthase to the cytoplasmic matrix for the physiological response in the cell. At this time, the cytoplasmic matrix ATP/ADP ratio is about 100 times that of the mitochondrial matrix ATP/ADP (Maldonado et al., 2017). Under the pathological condition, the function of ANT can be reversed and ATP can be injected into mitochondria for F1F0-ATP synthase hydrolysis. However, the synergistic change of the function of F1F0-ATP synthase and ANT does not occur at the same time, because the change of membrane potential causing the reversal of F1F0-ATP synthase function is greater than the decrease of membrane potential causing the reversal of ANT function. The critical value of membrane potential during functional transition is defined by reversing constant Erev-F1F0-ATPase and Erev-ANT, respectively. When the membrane potential is negatively polarized and less than Erev-F1F0-ATPase and Erev-ANT, the F1F0-ATP synthase synthesizes ATP, and ANT transports ATP from the mitochondria. When the membrane potential is depolarized, both greater than Erev-F1F0-ATPase and Erev-ANT, F1F0-ATP synthase hydrolyzes ATP, and ANT transports ATP from the cytoplasm to the mitochondria. When the membrane potential is between Erev-F1F0-ATPase and Erev-ANT, only the function of F1F-ATP synthetase shifts and the function of ANT does not change, that is, only when the membrane potential decreases slightly, F1F0-ATP synthase can hydrolyze mitochondrial ATP without affecting the cytoplasmic ATP content (Chinopoulos, 2011).

The mitochondrial membrane potential is an important index to observe early apoptosis. When the mitochondrial membrane potential decreases, the pro-apoptotic proteins located between the inner and outer membranes of the mitochondria are released into the cytoplasm, leading to apoptosis. The mitochondrial membrane potential of protein is related to mPTP, In the early stage of apoptosis, mPTP can allow molecules with relative molecular weight less than 1.5 × 10^3^ kDa to pass through, which leads to the decrease of mitochondrial membrane potential, the increase of mitochondrial membrane permeability and the release of pro-apoptotic proteins, which leads to apoptosis (Fall and Bennett, 1999).

Mitochondrial damage in any part of the brain can lead to insufficient mitochondrial energy, while migraine is a response to brain energy deficiency or oxidative stress levels exceeding antioxidant capacity (Zhang et al., 2011; Kowalska et al., 2021). Oxidative phosphorylation dysfunction, mitochondrial membrane potential changes, ROS production, and energy metabolism disorders may affect astrocytes. Astrocytes repair and protect cells in the nervous system. The role of astrocytes is to eliminate ROS and maintain the environment of extracellular ions and neurotransmitters, both of which are energy-dependent processes (Chen et al., 2003; Lian and Stringer, 2004). Energy consumption caused by mitochondrial dysfunction can damage the function of astrocytes, thus increasing the susceptibility of neurons to CSD.

### Translocator proteins

TSPO is a ubiquitous conserved protein located in the outer membrane of mitochondria. In the past few decades, the role of TSPO has been widely studied. It can form a complex with VDAC or ANT, has the function of transporting cholesterol during steroid production, and is also involved in regulating cell proliferation, apoptosis, and migration (Lin et al., 2014), as well as mitochondrial respiratory and oxidative stress. It has been reported that TSPO inhibits downstream mitochondrial autophagy by producing ROS to inhibit ubiquitin ligase parkin (PARK2)-induced protein ubiquitination (Gatliff et al., 2014). TSPO increases the level of ROS by regulating mitochondrial Ca^2+^ signal transduction, increasing the level of cytoplasm Ca^2+^, and activating NADPH oxidase (NOX). Downregulation of TSPO expression can reduce the level of ROS in hypoxia/reoxygenation cardiomyocytes and reduce oxidative stress, mitochondrial damage, and apoptosis (Meng et al., 2020).

**FIGURE 2:**
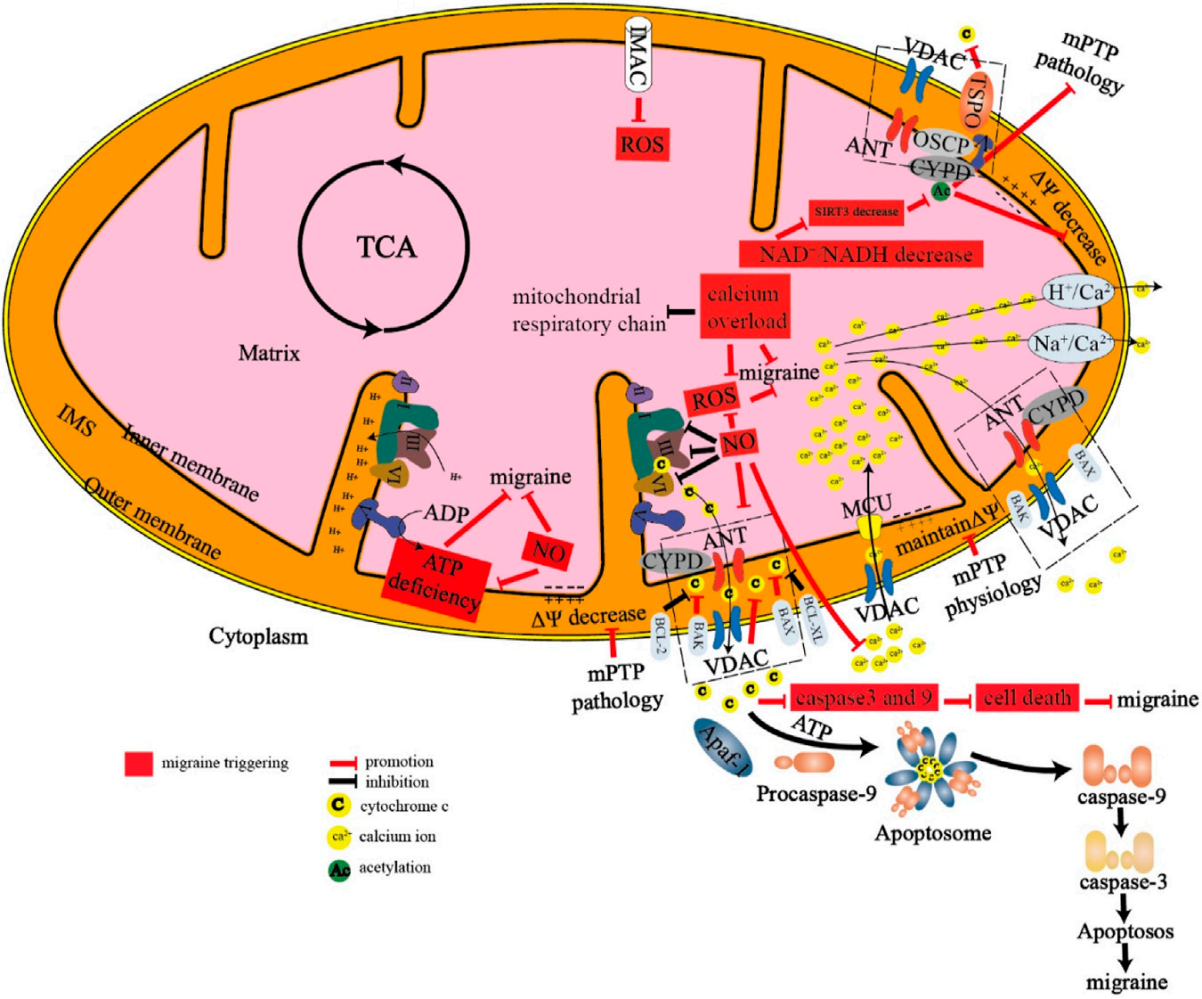
Mechanism of energy metabolism in mitochondria there are many proteins and protein channels on the inner membrane and outer membrane of mitochondria. When energy metabolism is abnormal, the following abnormalities will occur in mitochondria, which eventually lead to migraine. 1. mPTP and IMAC are pathologically open, resulting in the release of cytochrome C and reactive oxygen species. 2. A large number of calcium ions inflow through the MCU channel, resulting in calcium overload in the mitochondria. 3. The dysfunction of the mitochondrial respiratory chain leads to the production of reactive oxygen species and insufficient production of ATP, which will stimulate the production of nitric oxide. 4. The decrease of NAD+/NADH can lead to the change of SIRT3, CYPD, and other proteins, and finally lead to the decrease of mitochondrial membrane potential and the opening of mitochondrial permeability transition pore. 5. Abnormal proportion of pro-apoptotic proteins and anti-apoptotic proteins can lead to apoptosis and eventually lead to migraine.

TSPO knockout and the role of TSPO ligands, including TSPO/VDAC interactions, have demonstrated the effects of TSPO on gene expression and function (Yasin et al., 2017). The role of TSPO in metabolism is illustrated by studies showing that in TSPO knockout mice, TSPO knockout leads to altered mitochondrial energy metabolism, together with reduced oxygen consumption, mitochondrial membrane potential and ATP. TSPO also controls mitochondrial energy homeostasis by regulating fatty acid oxidation in steroidogenic cells (Lan et al., 2016). In addition, TSPO regulates autophagy by generating acute ROS and preventing PARK2 from completing protein ubiquitination (Gatliff et al., 2014). It has been reported that TSPO deregulates mitochondrial Ca^2+^ signaling, leading to an increase in cytoplasm Ca^2+^ levels, resulting in the activation of Ca^2+^-dependent NOX, which increases ROS levels (Gatliff et al., 2017). Mitophagy, a specific autophagic pathway, promotes the turnover of damaged mitochondria that are engulfed in autophagosomes through a lysosomal-dependent process. However, there is increasing evidence that a form of autophagic cell death is induced by oxidative stress (Ren et al., 2019). TSPO inhibits mitophagy and prevents the necessary ubiquitination of proteins. Recent studies have shown elevated levels of TSPO in the brain and/or spinal cord of patients with various chronic pain conditions (such as chronic low back pain, fibromyalgia, migraine and Gulf War illness) (Figure 2) (Alshelh et al., 2022).

mPTP, mitochondrial permeability transition pore; IMAC, inner membrane anion channel; ANT, adenine nucleotide translocase; VDAC, voltage-dependent anion channel; CYPD, Cyclophilin D; OSCP, oligomycin sensitivity-conferring protein; TSPO, translocator protein; MCU, mitochondrial calcium uniporter; NAD+, nicotinamide adenine dinucleotide; NADH, reduced nicotinamide adenine dinucleotide; SIRT3, Sirtuin 3.

## Oxidative phosphorylation inhibitor

Oxidative phosphorylation inhibitors mainly include oligomycin, carbonyl cyanide p-trifluoromethoxyphenylhydrazone (FCCP), rotenone, and antimycin A, which have different functions. Understanding their specific action sites is helpful for us to better understand and design the experiment.

### Oligomycin

Oligomycin is an inhibitor of oxidative phosphorylation in mammalian cells. It can effectively bind the functional subunit F0 of mitochondrial F1F0-ATP synthase and change the configuration of ATP synthase, thus inhibiting the proton flow from the mitochondrial intermembrane space back to the mitochondrial matrix. As a result, the synthesis of ATP is blocked, resulting in a lack of energy needed for biological metabolism, so oligomycin is highly toxic to mammals. However, it has also been shown that oligomycin can act as an inhibitor of tumor cell apoptosis. In mouse histiocytomas, oligomycin treatment reduced cellular ATP levels and significantly inhibited apoptosis, and also oligomycin inhibited caspase-1 and caspase-3 activities and loss of mitochondrial membrane potential, suggesting that the inhibition of apoptosis by oligomycin may be due to inhibition of ATP production, caspase activity and inhibition of mitochondrial depolarization (Singh and Khar, 2005). The change of cellular biological energy is closely related to migraine. The regulation of F1F0-ATP synthase that makes eukaryotic cells produce most of ATP is expected to be used in the treatment of migraine (Johnson et al., 2006). Mitochondria are key regulators of programmed cell death and energy metabolism, which means that mitochondria can be used as targets for the treatment of migraine.

### Carbonyl cyanide P-trifluoromethoxyphenylhydrazone

FCCP is a lipophilic weak acid, which can easily spread through the inner membrane of the mitochondria and enter the acidic mitochondrial intermembrane space, bringing the H+in the intermembrane space back to the mitochondria and releasing it into the matrix in an uncharged form, thus eliminating the H^+^ concentration gradient on both sides of the inner membrane of the mitochondria, resulting in the loss of the activated proton driving force of ATP synthase and the inability to synthesize ATP, thus uncoupling Oxidative phosphorylation, inhibition of oxidative phosphorylation (Monteiro et al., 2011). As an uncoupling agent of the electron transport chain and oxidative phosphorylation, FCCP is a proton carrier, which reduces the level of ATP by consuming ATP. However, if the concentration of FCCP is too high, it will not only reduce the production of ATP but also consume the ATP of the cell itself, resulting in cell death or changes in a survival state, which is not conducive to follow-up research but also makes the experimental results ineffective. Therefore, when using an Extracellular Flux Analyzer to determine the changes in cell bioenergy metabolism, the appropriate concentration of FCCP is very important.

### Rotenone

Rotenone is a fat-soluble substance extracted from the natural plant *Derris trifoliata* Lour and has been widely used as an insecticide in agricultural production since the 1940s. It has strong fat solubility and can easily pass through the blood-brain barrier (BBB). Into the brain mitochondria, selectively block the role of iron-sulfur cluster N2 and CoQ to inhibit the mitochondrial respiratory chain, resulting in cytotoxicity, resulting in apoptosis. Mitochondria are the largest iron metabolizing organelles in the cell and are mainly responsible for the synthesis of heme and iron-sulfur clusters. Heme and iron-sulfur clusters are two important cofactors involved in the synthesis and repair of DNA, protein synthesis and folding, tricarboxylic acid cycle, and the normal conduct of the mitochondrial electron transport chain. Disruption of iron metabolism in mitochondria can seriously affect the iron metabolism and energy metabolism of the whole cell, thus affecting the function of mitochondria and leading to the development of various diseases (Wachnowsky et al., 2018). Studies have shown that rotenone has a high affinity with mitochondria and can selectively inhibit mitochondrial complex I, which in turn affects mitochondrial function and cell survival (Xiong et al., 2012). The loss of mitochondrial membrane potential plays an important role in the process of apoptosis. Rotenone significantly reduces the mitochondrial membrane potential and ATP content. Mitochondrial membrane potential is an important index to reflect the function of mitochondria. The change of mitochondrial membrane potential affects the function of the proton pump and then affects the production of ATP.

### Antimycin A

Antimycin A, an inhibitor of mitochondrial complex III, is a bactericidal antibiotic. Its mechanism includes the inhibition of electron transport between nicotinamide adenine dinucleotide (NADH) oxidase and mitochondrial cytochrome bc1 (Ransac and Mazat, 2010). The inhibition of electron transport in mitochondria will lead to the collapse of the proton gradient across the mitochondrial inner membrane, thus destroying the mitochondrial membrane potential; this inhibition also leads to the formation of ROS.

## Relationship between tricarboxylic acid cycle and migraine

**FIGURE 3:**
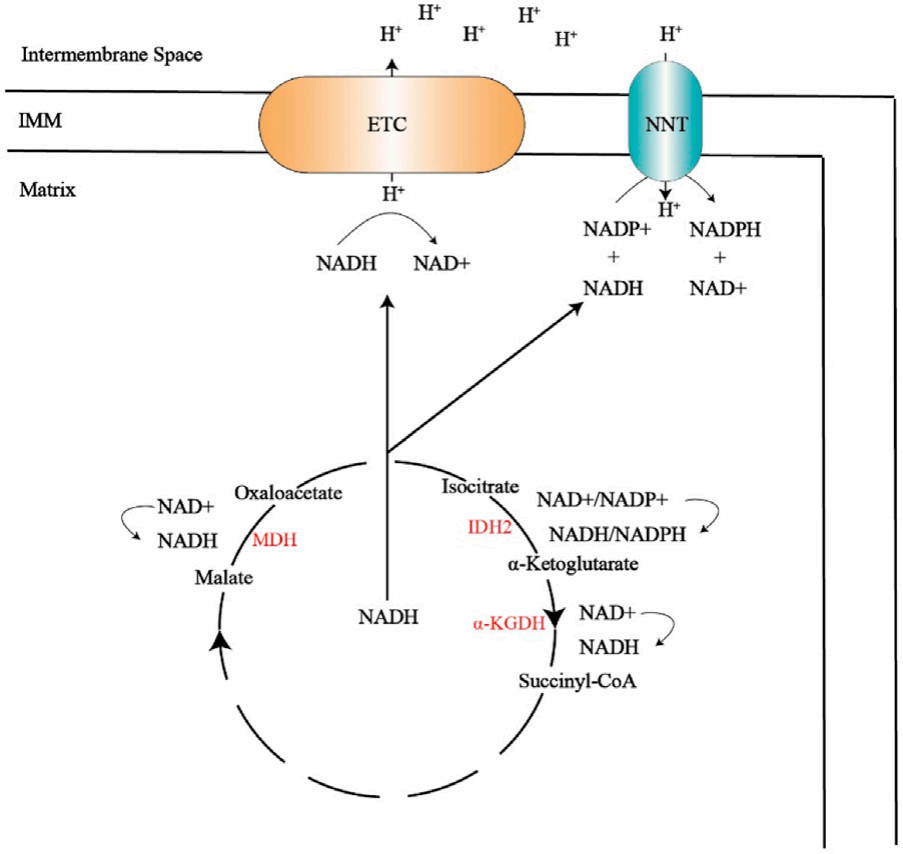
The tricarboxylic acid cycle produces NADH, which provides NADH for the electron transport chain and NNT. The electron transport chain can transfer hydrogen ions to the intermembrane space, and the hydrogen ions located in the membrane gap pass through NNT, resulting in antioxidant NADPH.

MDH, malate dehydrogenase; IDH2, Isocitrate dehydrogenase 2; α-KGDH, α-ketoglutarate dehydrogenase; IMM, inner mitochondrial membrane; ETC, electron transport chain; NNT,nicotinamide nucleotide transhydrogenase; NADP+, nicotinamide adenine dinucleotide phosphate; NADPH, reduced form of nicotinamide adenine dinucleotide phosphate.

Mitochondria are the center of cellular energy metabolism, and the TCA cycle in mitochondria is a common metabolic pathway in aerobic organisms. The main function of the TCA cycle is to produce reducing equivalents, such as NADH and FADH_2_ (produced by succinate dehydrogenase). NADH and FADH_2_ can transfer electrons to, ETC., to drive oxidative phosphorylation and produce ATP. The TCA cycle consists of eight enzymes, namely, citrate synthase (CS), aconitase (ACO2), isocitrate dehydrogenase (IDH), α-ketoglutarate dehydrogenase (α-KGDH), succinyl CoA synthetase (SCS), succinate dehydrogenase (SDH), fumarase hydratase (FH) and malate dehydrogenase (MDH). Related studies evaluated the activity of platelet mitochondrial enzymes in patients with migraine with or without aura, and found that complex I, CS, and complex Ⅳ were damaged in migraine patients (Sangiorgi et al., 1994). Biochemical studies have shown that the level of lactic acid in cerebrospinal fluid of patients with migraine attacks is significantly higher than that in the intermittent period because lactic acidosis indicates the disturbance of pyruvate utilization in the TCA cycle. The finding that migraine patients show signs of lactic acidosis, thus suggesting abnormalities in the functioning of the TCA, has led researchers to further investigate the TCA, for example, for pyruvate, SDH, nicotinamide adenine dinucleotide cytochrome c reductase, succinate cytochrome c reductase, NADH dehydrogenase and CS, in order to investigate the relationship between migraine and the TCA (Figure 3) (Stuart and Griffiths, 2012). Under physiological conditions, the TCA produces NADH, and NNT uses NADH as a substrate to produce nicotinamide adenine dinucleotide phosphate (NADPH). This reaction is energetically favored because the transhydrogenation between NADH and NADPH is coupled to the proton gradient across the IMM. NNT and NADPH play an important role in the scavenging of ROS, so the TCA is essential for maintaining the body’s antioxidant levels. However, in pathological conditions, NNT depletes NADPH and regenerates NADH to produce ATP to satisfy the body’s energy requirements (Bertero and Maack, 2018). The expression of antioxidant enzymes SOD2, catalase, and glutathione peroxidase 3 (GPx-3) decreased significantly in IDH and NNT knockout mice (Lee et al., 2020). CSD can lead to a sharp decrease in NADH content, the destruction of the proton gradient, and the damage of NNT to antioxidant defense (Borkum, 2021). Valproic acid (VPA) and its salts are widely used in migraine, the metabolism of VPA is complex and continues to be studied. Known pathways of VPA metabolism is β-oxidation in the tricarboxylic acid cycle (acetylation). This could also prove that there is a correlation between migraine and the TCA (Figure 3) (Shnayder et al., 2023).

## Relationship between reactive oxygen species and migraine

Mitochondria are the main sites for the production of ROS, and mROS mainly comes from electron leakage of oxidative phosphorylation of mitochondria. ROS are produced by extra electrons obtained by O_2_, including superoxide anions (O_2-_), hydrogen peroxide (H_2_O_2_), and hydroxyl radicals (OH), among which H_2_O_2_ is the most important ROS signal molecule in cells. ROS are a series of highly active superoxide anion radicals formed by one molecule O_2_ receiving one electron (Sheng et al., 2014).

In the process of electron transfer along the mitochondrial, ETC, peroxides and other ROS are formed, collectively referred to as mitochondrial-derived reactive oxygen species (mROS). Complex I and III can produce mROS, when the NADH/NAD^+^ ratio is high, the TCA cycle dehydrogenases also generate high amounts of ROS. When NAD^+^ is not available, oxygen becomes the default electron acceptor, producing superoxide and hydrogen peroxide. *In vitro* study showed that NAD^+^ administration had protective effects on hypoxia-induced neuroinflammation and mitochondrial damage, and ROS production in BV2 microglia through the Sirt1/PGC-1α pathway (Zhao et al., 2021). ROS produced by abnormal mitochondrial function have been linked to migraine. Migraineurs have an impaired metabolic capacity with increased ROS production. NTG induced more ROS in the low glucose condition than in the high glucose condition. The mitochondrial dysfunction detected by Seahorse may explain the increase in ROS. Inhibition of ROS may have therapeutic benefits in the prevention of migraine. Antioxidants may become a potential drug for migraine treatment (Li et al., 2022). Under normal physiological conditions, oxygen free radicals are catalyzed by antioxidant factors such as SOD and glutathione peroxidase (GPX) to form water, which keeps intracellular ROS at a harmless low level. When the body is under oxidative stress, a large number of ROS will be produced in the mitochondria. These oxygen free radicals can unselectively damage various components of the cell, and intracellular autophagy can reduce the secondary harm of the damaging substances and delay cell death caused by stress. ROS can reduce the level of ROS by inducing autophagy, but excessive ROS can induce excessive autophagy and induce apoptosis (Yu et al., 2006; Castino et al., 2010). When the production of mROS exceeds the antioxidant capacity of the body, oxidative stress occurs, which consumes reduced glutathione (GSH) and damages cellular components such as proteins, lipids, DNA, and sugars, resulting in oxidative damage and mutation of mitochondrial DNA and increased permeability of mPTP. It further leads to the decrease of mitochondrial membrane potential, the decrease of the amount of ATP produced by cells, and the acceleration of mitochondrial apoptosis, which leads to cell apoptosis (Kalogeris et al., 2014). The increase of permeability of mPTP can further promote the accumulation of mROS, which is called ROS-induced ROS release (RIRR) (Zorov et al., 2014). Studies have shown that the content of malondialdehyde (MDA) in blood samples of migraine patients increases (Bernecker et al., 2011). MDA is a lipid peroxide and a marker of oxidative stress. Lipid peroxidation not only increases the production of mROS but also destroys the integrity of the mitochondrial membrane and opens mPTP. Opening of mPTP is an important step in the mechanism of necrosis and apoptosis (Ma et al., 2017), When mPTP is opened, its large conductance can lead to rapid swelling due to the osmotic pressure of matrix solute, the rupture of the mitochondrial outer membrane and the disintegration of mitochondrial membrane potential (Sanderson et al., 2013). mROS plays an important role in regulating some normal functions of the body and tissue. If mROS increases, without being decomposed by cells in time, it will reduce the activity of the respiratory chain complex, thus reducing the function of mitochondria, affecting oxidative phosphorylation and reducing ATP synthesis, while insufficient synthesis of ATP will further damage mitochondria and induce more mROS. Finally, excessive mROS can not be eliminated in time, resulting in accumulation. Thus damage to mitochondria, cell metabolism disorder, can not work properly, or even apoptosis, causing migraine (Sparaco et al., 2006). Under physiological conditions, the increase of Ca^2+^ in mitochondria will promote the production of ATP, but in pathological conditions, the increase of Ca^2+^ in mitochondria will promote the production of ROS (Brookes et al., 2004). It has been found that CSD can induce the formation of ROS in the cortex, meninges, and trigeminal ganglia. ROS can directly activate painful electrophysiology and promote peptidergic nerves to release migraine mediator CGRP. CGRP can further promote the sensitization and inflammation of meningeal neurons related to oxidative stress. Therefore, both direct and indirect effects of ROS can lead to migraine. Antioxidants and ROS scavengers can reduce the pain caused by ROS (Shatillo et al., 2013).

### Oxidative stress marker

It was found that advanced oxidation protein products (AOPP) were significantly decreased in migraine patients treated with CGRP receptor antagonists (De Luca et al., 2021). AOPP is not only considered a marker of protein damage caused by oxidative stress, but also an important mediator of oxidative stress and inflammation (Wei et al., 2009). 8-hydroxy-2 deoxyguanosine (8-OHdG) is an oxidative adduct produced by free radicals such as hydroxyl radical and singlet oxygen, which attacks the eighth carbon atom of guanine base in the DNA molecule. It is related to oxidative stress damage in cerebrovascular diseases. The higher the 8-OHdG level, the more serious the oxidative stress damage (Li et al., 2013). 8-OHdG reflects the oxidative damage of the nucleus and mitochondrial DNA caused by free radicals. It was found that the serum 8-OHdG of migraine patients was significantly higher than that of healthy controls (Geyik et al., 2016).

### Active oxygen scavenger

**FIGURE 4:**
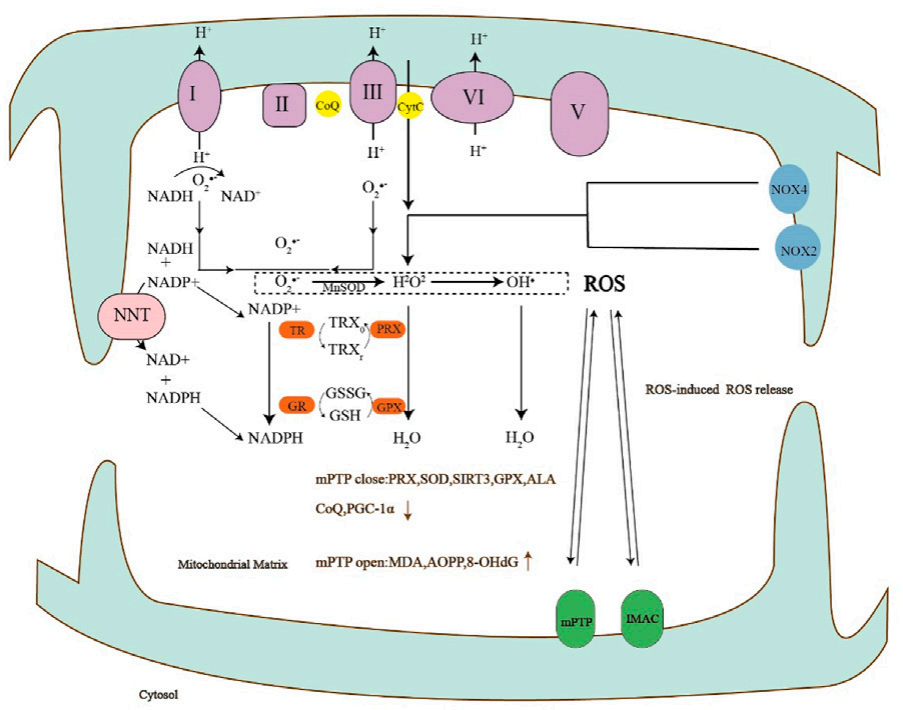
The production of reactive oxygen species can lead to the opening of mPTP, the opening of mPTP can increase the markers of oxidative stress, and the close of mPTP can lead to the decrease of antioxidants.

I-V, complexes I-V of the respiratory chain; CoQ, Coenzyme Q; CytC, Cytochrome c; NOX, NADPH oxidases; TR, thioredoxin reductase; PRX, peroxiredoxin; GR, glutathione reductase; GPX, glutathione peroxidase; TRX_r_/TRX_o_, reduced/oxidized TRX; GSH, reduced glutathione; GSSG, oxidized glutathione; SOD, Superoxide dismutase; ALA, alpha-thioctic acid; PGC-1α, Peroxisome proliferator-activated receptor-gamma co-activator-1alpha; MDA, malondialdehyde; AOPP, Advanced oxidation protein product; 8-OHdG, 8-hydroxy-2-deoxyguanosine.

mROS scavenging mainly depends on three kinds of superoxide dismutase and antioxidant proteins, of which the highest level is antioxidant peroxidase (PRX), while PRX3 is responsible for the degradation of most of the intracellular H_2_O_2_. Some studies have shown that the reduction of H_2_O_2_ degradation after PRX1 phosphorylation leads to the local accumulation of H_2_O_2_ into the cytoplasm, thus activating the growth factor-dependent signal pathway (Woo et al., 2010). Superoxide dismutase (SOD) is an endogenous antioxidant that scavenges oxygen free radicals. Studies have found that SOD activity continues to decrease in migraine patients (Neri et al., 2015). The clearance of ROS in mitochondria is mainly degraded by SOD2. SOD2 is the only antioxidant enzyme in mitochondria that can catalyze ROS to hydrogen peroxide. The activity of the SOD2 enzyme was mainly affected by its acetylation level. However, the acetylation level of mitochondrial proteins is mainly regulated by SIRT3. SIRT3 can enhance the activity of oxygen-free radical scavenging enzymes, reduce the level of ROS in mitochondria and reduce oxidative stress damage. At present, three kinds of mitochondrial deacetylase have been found, which are SIRT3, SIRT4, and SIRT5. SIRT3 is the strongest deacetylase in mitochondria. SIRT3 regulates the level of intracellular ROS by regulating the acetylation level of SOD2. More importantly, SIRT3 can directly bind to SOD2, resulting in the deacetylation of SOD2 and enhancing the activity of SOD2, which plays an important role in regulating the homeostasis of ROS in mitochondria (Giralt and Villarroya, 2012). Most of the antioxidation of ROS in the cytoplasm is carried out through GPX. Glutathione can be dehydrogenated to oxidized glutathione (GSSG), which can be reduced to GSH by NADPH under the action of glutathione reductase (GR). GPX is one of the main enzymes that catalyze the oxidation of reduced glutathione in the glutathione redox cycle. GPX can not only specifically catalyze the reaction of GSH with ROS to form GSSG, protect the biofilm from ROS damage, and maintain the normal function of cells (Yin et al., 2016). ALA, also known as alpha-lipoic acid, is an eight-carbon sulfur compound, which has the functions of water-soluble and fat-soluble antioxidants. It can reduce oxidative stress directly (by removing active substances) and indirectly (by chelating into metal ions). As a coenzyme, ALA also plays an important role in energy metabolism (Müller and Krieglstein, 1995; Packer et al., 1995; Bast and Haenen, 2003). Riboflavin may play an important role in migraine prevention by participating in antioxidant and anti-inflammatory responses caused by mitochondrial dysfunction (Fila et al., 2021). Coenzyme Q10 (CoQ10) participates in many cellular redox reactions and plays an important role in bioenergetics and antioxidant defense. It can associate mitochondrial function with energy production and oxidative stress. Some studies have found that CoQ10 can play a role in the treatment of migraine (Parohan et al., 2020). Peroxisome proliferator-activated receptor-gamma coactivator 1 alpha (PGC-1α) is a transcriptional co-upregulation factor, which is highly expressed in the brain, heart, skeletal muscle, and other tissues. It plays a central role in controlling mitochondrial function and mitochondrial biogenesis and is a known positive regulator of mitochondrial function and oxidative metabolism (Fernandez-Marcos and Auwerx, 2011; Wang et al., 2015). It was found that repeated infusion of inflammatory soup into the dura mater could decrease the expression of PGC-1α in the trigeminal nucleus caudalis (TNC) in SD rats (Figure 4) (Liang et al., 2021).

### Relationship between NO and energy metabolism of migraine

NO is a kind of bioactive molecule existing in various tissues of organisms. It has protective effects such as dilating blood vessels and improving local blood circulation. Some studies have found that NO is a key factor in migraine and other headaches. It is considered to be the trigger point of primary headaches and plays a key role in the pathogenesis of migraine (Olesen, 2008). Studies have shown that oxidative stress levels are elevated during migraine attacks and decreased during the intermittent period (Borkum, 2021). As a result, most of the biological markers related to oxidative stress, such as some heavy metals (iron ions) and NO, have corresponding expression changes in different periods of migraine. The levels of these biomarkers related to oxidative stress can fluctuate significantly due to migraine attack frequency, attack cycle, aura, or not (Togha et al., 2019; Fila et al., 2021). NO is involved in the vascular mechanism induced by CSD (Pradhan et al., 2018). In the cat model of migraine induced by nitroglycerin, compared with the control group, CSD significantly enhanced the release of NO. Experimental studies have also shown that the changes in neurotransmitters and vasoactive substances caused by CSD play an important role in migraine attacks. CSD can cause the release of NO and the content of vasoactive peptides such as CGRP in plasma (Read et al., 2000). Many experimental studies and clinical drugs have confirmed that the pathogenesis of migraine is closely related to the vasomotor state, that is, there are many metabolic disorders of vasoactive neuropeptides or neurotransmitters during the attack of migraine. These evidences show that NO is closely related to migraine, and the production of NO causes migraine. A high concentration of NO can significantly inhibit the activity of many components in the mitochondrial respiratory chain, resulting in the damage of the mitochondrial respiratory chain and the decrease of ATP production, resulting in neuronal dysfunction and migraine. NO can modify the sulfhydryl group, heme prosthetic group, and iron-sulfur center of some proteins through biofilms such as cell membrane and mitochondrial membrane, and can directly regulate the binding and release of oxygen and heme. NO controls the oxygen supply to mitochondria in this way. A high concentration of NO could significantly inhibit the activities of many components of the mitochondrial respiratory chain, such as the binding site of cytochrome oxidase to oxygen and NADH dehydrogenase (Nisoli et al., 2003). NO and carbon monoxide (CO) have shared functions as they both bind to haemoglobin and inhibit the oxygen transport system. They also inhibit mitochondrial oxidative phosphorylation by competitive binding to cytochrome c oxidase. NO can bind both oxidised and reduced cytochrome c oxidase, migraine can occur through all of the above pathways (Arngrim et al., 2014). Under the action of NO, cells can control the release of Cyt-c by regulating the opening of mPTP. Generally speaking, in the presence of a high dose of NO, the opening of mPTP increases, and the mitochondrial membrane potential decreases, which leads to the increase of Cyt-c release (Brookes et al., 1999). Complex IV in the mitochondrial respiratory chain, which has been shown to bind NO, may reduce NO activity if the vasculature contains sufficient complex IV (Leao, 1947; Torres et al., 1995), thereby preventing vasodilation. Modification of the complex IV:cyt-c ratio increases the susceptibility of endothelial cells to apoptosis. Inhibition of mitochondrial protein synthesis by chloramphenicol results in an alteration of the complex IV:cyt-c ratio in mitochondria. This leads to an increase in the amount of free cyt-c in the mitochondrial intermembrane space. There is also an alteration in mitochondrial bioenergetics and ROS production. These factors then make the cell more susceptible to NO-induced apoptosis (Ramachandran et al., 2002).

Exogenous NO treatment significantly increased the content of ROS in mitochondria and apoptosis, and the content of ROS in mitochondria of neurons pretreated with NO scavenger hemoglobin (Hb) was similar to that of normal cells. This shows that NO can increase the content of ROS in mitochondria, which leads to apoptosis. In addition, high-dose NO can inhibit the enzyme activity of the mitochondrial respiratory chain, which will increase the electron leakage of mitochondria and increase the production of endogenous superoxide anions in mitochondria. Superoxide anion can react quickly with NO in mitochondria to form peroxynitrite (ONOO^−^) with greater toxicity and pro-apoptotic effect. The generated ONOO^−^ can directly damage the DNA of cells and induce apoptosis, and it can also stimulate p53 due to DNA damage, resulting in the decrease of Bcl-xl and the increase of Bax, resulting in the occurrence of apoptosis (Singh and Dikshit, 2007).

Studies have shown that nitroglycerin can be converted into NO *in vivo*, thus activating the TRPA1 channel to produce oxidative stress (Wenzl et al., 2009). The activation of the TRPA1 channel in trigeminal ganglion neurons can promote the production of ROS, while ROS can further activate trigeminal ganglion neurons to form a positive feedback circuit and increase the release of CGRP.

## Relationship between CGRP and energy metabolism of migraine

As a multifunctional neuropeptide, CGRP plays an important role in the pathophysiology of migraine by regulating neurogenic inflammation and nociceptive signal input (Russell et al., 2014; Russo, 2015). It has been found that ROS promotes CGRP production in a migraine rat model of CSD caused by Kcl stimulation, which allows ROS to reverse the reduction of cortical sensitivity to CSD after CGRP inhibition (Jiang et al., 2019). TRPA1 channel is a non-selective cation channel expressed in sensory nerve endings, which is widely distributed in nerves and non-nerve cells. Intracellular and extracellular Ca^2+^ is not only a key regulator of the TRP channel but also an important ion for activating and regulating the activity of the TRPA1 channel. The regulation of Ca^2+^ in TRPA1 is dependent on calmodulin (CaM). CaM binds to TRPA1 to form calcium sensitive channel complex (Hasan et al., 2017). Activation of TRPA1 channels increases intracellular calcium levels, similar to L-type voltage-gated calcium channels (Kowalska et al., 2021). VGCC are classified according to their electrophysiological and pharmacological properties. The activation threshold of P/Q (Cav2.1), R (Cav2.3), L (Cav1.1-Cav1.4), and N(Cav2.2)-type VGCC is higher than that of the T (Cav3.1-CaV3.3) channel. Although L-type and T-type VGCC are widely distributed in many cell types, P/Q, N-and R-type channels are mainly limited to neurons. P/Q-type and N-type calcium channels are located in neurons, and L-type calcium channels are mainly found in the trigeminal ganglion and spinal trigeminal nucleus of rats. CaV2.1 channel is a calcium channel located in the presynaptic membrane and plays an important role in the communication between neurons by controlling the release of neurotransmitters. The mutation of the CACNA1A gene leads to the increase of CaV2.1 activation, which in turn leads to the increase of intracellular Ca^2+^. Except for CaV2.1, other VGCCs may play a role in the pathogenesis of migraine (Nanou and Catterall, 2018). Although presynaptic CaV2 channels may drive the release of CGRP associated with migraine, high voltage activated and typical postsynaptic CaV1 channels and low voltage activated CaV3 channels have also been found to regulate CGRP release in the trigeminal ganglion, which can be proved by drug blocking experiments (Amrutkar et al., 2011). TRPA1 channel has received extensive attention in migraine studies because it is activated by a series of endogenous and exogenous stimuli that may be associated with migraine. These substances include ROS and nitrogen (Nassini et al., 2014). ROS can activate TRP channels and promote the release of CGRP from sensory nerve endings. The function of CGRP in various processes can also be explored by TRP (Dussor et al., 2014; Russell et al., 2014; Veldhuis et al., 2015). H_2_O_2_ is often used as an inducer of oxidative damage to cells. By inducing the production of ROS and causing inflammation, H_2_O_2_ is involved in the pathogenesis of many nervous system diseases (Lacraz et al., 2009). H_2_O_2_ can activate sensory neurons through the cysteine group in the TRPA1 channel, while H_2_O_2_ can promote the production of CGRP (Shatillo et al., 2013). TRPV1 and TRPA1 channels are expressed by peptidergic trigeminal neurons, which synthesize and store the main migraine mediator CGRP (Andersson et al., 2008). The increase of oxidative stress in trigeminal ganglion leads to a significant increase in CGRP level in trigeminal ganglion (Marone et al., 2018; Messlinger et al., 2020), which is also closely related to the pathophysiology of migraine.

**FIGURE 5:**
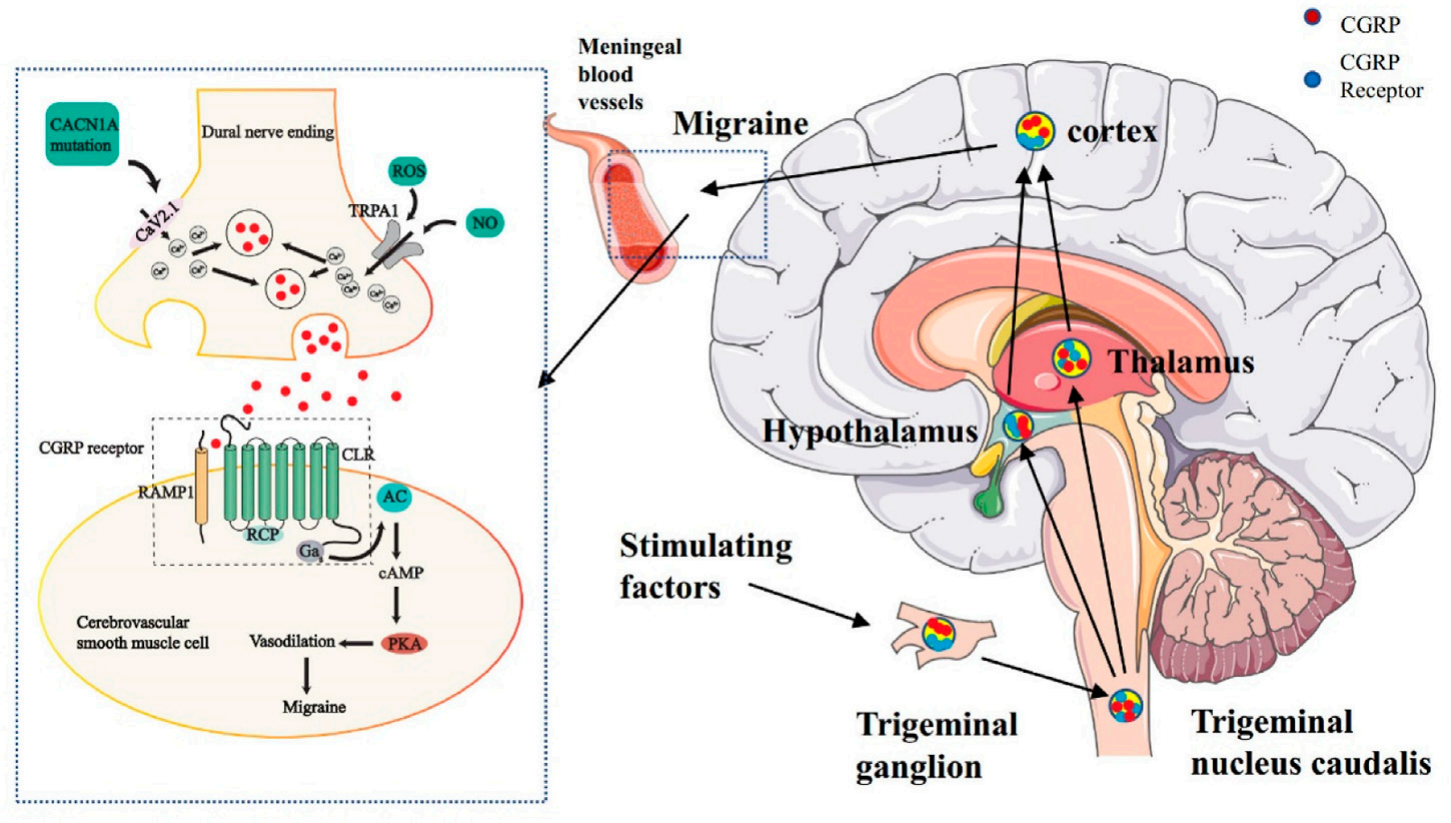
The dysfunction of the trigeminovascular system and energy metabolism can lead to migraine. The primary afferent nerve of the trigeminal nerve innervated the pia mater and dural meningeal vessels. Its efferent projection fibers connect with secondary neurons in the TNC of the brainstem. The nerve fibers of TNC project to the thalamus and then rise further to connect with the higher cortical area. At the junction of nerve endings and vascular smooth muscle, ROS and NO jointly activate the TRPA1 channel, which leads to calcium influx, stimulates CGRP release, binds to the CGRP receptor of vascular smooth muscle, and then leads to vasodilation and migraine.

It is generally admitted that activation and sensitization of primary afferent nociceptors that innervate the dural and meningeal vasculature trigger both CGRP-induced vasodilatation and neurogenic inflammation. Pain signals pass through the TNC, which relays signals to higher-order neurons in the thalamus and cortex. Central and peripheral sensitization may contribute to the maintenance of pain signals and predispose them to future migraine attacks (Takayama et al., 2019). Melo-Carrillo and colleagues further demonstrated that CSD leads to sensitization of central trigeminal nociceptive neurons. That study also found that systemic administration of an anti- CGRP antibody, used in migraine prophylaxis, inhibited the development of both the activation and sensitization of high threshold trigeminal neurons following CSD, further substantiating the link between CSD, meningeal nociception, and migraine headache (Figure 5) (Carneiro-Nascimento and Levy, 2022).

Gas, guanine nucleotide-binding proteins alpha s; AC, adenylate cyclase; cAMP, Adenosine monophosphate; PKA, protein kinase A; CLR, calcitonin receptor-like receptor; RAMP1, receptor activity-modifying protein 1; RCP, receptor component protein.

## Conclusion

In the past few years, we have made great progress in understanding the mechanism of energy metabolism in migraine. KCL-induced CSD and nitroglycerin-induced cerebral vasodilation are commonly used migraine models, which can cause disorders of energy metabolism and lead to oxidative phosphorylation and TCA dysfunction. The dysfunction of oxidative phosphorylation is mainly related to calcium ions, mPTP, mitochondrial membrane potential, CYPD, OSCP, TSPO, and the Bcl-2 family. Oxidative phosphorylation inhibitors are also the focus of attention. In some studies, oxidative phosphorylation inhibitors can be used to specifically inhibit complex I, complex II, complex III, and complex IV, to observe the corresponding affected proteins.

The relationship between ROS and energy metabolism is also very close. Too much ROS will lead to oxidative stress, which will promote the opening of mPTP, and then affect the expression of pro-apoptotic and anti-apoptotic proteins of the Bcl-2 family. ROS and CGRP, TRPA1, and TRPV1 can interact to cause migraine.
